# Growth of *Rhodococcus* sp. strain BCP1 on gaseous *n*-alkanes: new metabolic insights and transcriptional analysis of two soluble di-iron monooxygenase genes

**DOI:** 10.3389/fmicb.2015.00393

**Published:** 2015-05-12

**Authors:** Martina Cappelletti, Alessandro Presentato, Giorgio Milazzo, Raymond J. Turner, Stefano Fedi, Dario Frascari, Davide Zannoni

**Affiliations:** ^1^Department of Pharmacy and Biotechnology, University of BolognaBologna, Italy; ^2^Department of Biological Sciences, University of CalgaryCalgary, Alberta, Canada; ^3^Department of Civil, Chemical, Environmental and Materials Engineering, University of BolognaBologna, Italy

**Keywords:** *Rhodococcus* sp. strain BCP1, soluble di-iron monooxygenase, propane and *n*-butane oxidation, gaseous *n*-alkanes, monooxygenase gene expression

## Abstract

*Rhodococcus* sp. strain BCP1 was initially isolated for its ability to grow on gaseous *n*-alkanes, which act as inducers for the co-metabolic degradation of low-chlorinated compounds. Here, both molecular and metabolic features of BCP1 cells grown on gaseous and short-chain *n*-alkanes (up to *n*-heptane) were examined in detail. We show that propane metabolism generated terminal and sub-terminal oxidation products such as 1- and 2-propanol, whereas 1-butanol was the only terminal oxidation product detected from *n*-butane metabolism. Two gene clusters, *prmABCD* and *smoABCD*—coding for Soluble Di-Iron Monooxgenases (SDIMOs) involved in gaseous *n*-alkanes oxidation—were detected in the BCP1 genome. By means of Reverse Transcriptase-quantitative PCR (RT-qPCR) analysis, a set of substrates inducing the expression of the *sdimo* genes in BCP1 were assessed as well as their transcriptional repression in the presence of sugars, organic acids, or during the cell growth on rich medium (Luria–Bertani broth). The transcriptional start sites of both the *sdimo* gene clusters were identified by means of primer extension experiments. Finally, proteomic studies revealed changes in the protein pattern induced by growth on gaseous- (*n*-butane) and/or liquid (*n*-hexane) short-chain *n*-alkanes as compared to growth on succinate. Among the differently expressed protein spots, two chaperonins and an isocytrate lyase were identified along with oxidoreductases involved in oxidation reactions downstream of the initial monooxygenase reaction step.

## Introduction

Gaseous *n*-alkanes ranging from ethane (C_2_) to *n*-butane (C_4_) are categorized as non-methane hydrocarbons. Volatile aliphatic *n*-alkanes comprise of gaseous and liquid *n*-alkanes up to *n*-heptane (C_7_). Increasing concentrations of these *n*-alkanes in the atmosphere is problematic because of their contributions to ozone enhancement and photochemical smog formation (Chan et al., 2006; Shennan, 2006). From a biotechnological perspective, gaseous and short-chain *n*-alkanes (up to C_7_) are inexpensive carbon sources for microbial growth and bio-production of biosurfactants and polyhydroxyalkanoates (PHAs) (Lageveen et al., 1988; Philp et al., 2002).

Aerobic bacterial metabolism of gaseous- and short-chain liquid *n*-alkanes has received little attention compared to that of methane (C_1_) and/or longer chain *n*-alkanes (C >12) (Kotani et al., 2006). Our present knowledge on the metabolism of short-chain *n*-alkanes is based on studies in Gram-negative bacteria, when the most common bacterial species isolated from natural habitats that utilize gaseous *n*-alkanes are Gram-positive strains belonging to the CMNR group (including *Corynebacterium, Nocardia, Mycobacterium*, and *Rhodococcus* genera). *Thauera butanivorans* (formerly called “*Pseudomonas butanovora*”) is the best characterized Gram-negative microorganism able to metabolize gaseous *n*-alkanes, while the majority of data on gaseous *n*-alkane microbial growth is based upon the microbial ability to utilize putative metabolic intermediates such as propanal, acetone, and acetal (Ashraf et al., 1994; Arp, 1999; Kulikova and Bezborodov, 2001; Sluis et al., 2002; Kotani et al., 2003; Dubbels et al., 2007; Cooley et al., 2009).

Bacteria can utilize *n*-alkanes as growth substrates under aerobic conditions through the use of a monooxygenase reaction. The insertion of one oxygen atom from dioxygen (O_2_) into the alkane is catalyzed by iron- or copper-containing monooxygenase (MO) enzymes (Hamamura et al., 1999; van Beilen and Funhoff, 2007). Among these, Soluble Di-Iron Monooxygenases (SDIMOs) are multi-component enzymes that catalyze the initial oxidation of low molecular weight alkanes and alkenes as well as chlorinated solvents, aromatic hydrocarbons and cyclic ethers (Coleman et al., 2006). Recent studies have classified these enzymes into different groups based on gene organization, substrate specificity, and sequence similarity (Coleman et al., 2006, 2011). Since SDIMOs are responsible for several bioremediation and biocatalytic processes, widening our knowledge on their genetic diversity and on the growth conditions inducing their expression is of crucial importance for microbial biotechnology.

The Gram-positive soil microorganism, *Rhodococcus* sp. strain BCP1, was initially described for its ability to grow on gaseous *n*-alkanes as inducing condition for low chlorinated compound co-metabolism (Frascari et al., 2005, 2006, 2007; Cappelletti et al., 2012; Ciavarelli et al., 2012). In a previous study we have shown that in BCP1 cells, the expression of a gene (*alkB*) encoding an AlkB-like monooxygenase was induced by growth on liquid *n*-alkanes ranging from *n*-hexane (C_6_) to *n*-eicosane (C_20_) (Cappelletti et al., 2011). Here, we focused our attention on the ability of BCP1 to grow on short-chain *n*-alkanes (<C_7_) and the genetic and metabolic aspects involved. To this end, we analyzed gaseous *n*-alkane oxidation products and studied O_2_ consumption activity induced by short-chain *n*-alkanes (C_1_–C_7_) and their putative metabolic intermediates. Furthermore, two gene clusters coding for two different SDIMOs were found in the genome of BCP1 and named *prm* and *smo* according to similar operons present in the database. Both *prm* and *smo* were co-transcribed as single polycistronic units. The range of *n*-alkanes inducing their expression and their transcriptional start sites were defined, along with the effects of alternative carbon sources on their transcriptional induction. Finally, proteomic analysis provided useful insights on enzymes of BCP1 acting downstream of the initial oxidation step of gaseous (*n*-butane) and liquid (*n*-hexane) *n*-alkanes.

## Materials and methods

### Bacterial strains, growth media, and culture conditions

*Rhodococcus* sp. strain BCP1 was grown for 2 days on 10 mL of Luria–Bertani (here indicated as LB) for the genomic DNA purification and for the precultures preparation. The growth of BCP1 on *n*-alkanes and their metabolic intermediates was conducted in 119-mL bottles containing 40 mL of mineral medium (MM) [composed of (in micromoles) (NH_4_)_2_SO_4_, 797; MgSO_4_·7H_2_O, 244; CaCl_2_, 132; K_2_HPO_4_, 8900; NaH_2_PO_4_ · H_2_O, 5355; FeSO_4_·7H_2_O, 22.6; NaNO_3_, 9000; MnCl_2_·4H_2_O, 1.52; ZnSO_4_·7H_2_O, 0.510; H_3_BO_3_, 1.00; Na_2_MoO_4_·2H_2_O, 0.450; NiCl_2_·2H_2_O, 0.144; CuCl_2_·2H_2_O, 0.100; CoCl_2_·6H_2_O, 0.100] (Frascari et al., 2006). Each bottle was inoculated with 100 μL of the BCP1 preculture and then sealed with butyl rubber stoppers and aluminum crimp seals. BCP1 cultures were incubated at 30°C with shaking at 150 rpm. In the growth experiments, for each substrate, three bottles were inoculated. Gaseous *n*-alkanes were added at the final aqueous concentrations of 180 μM. Liquid *n*-alkanes, alcohols, butyric aldehyde and butyric acid (as sodium salt) were supplied at the final concentration of 0.1% (v/v). Succinate was supplied at the final concentration of 0.1% (w/v). To determine the generation time, an aliquot of each culture of BCP1 was spread onto LB plates every 12 h and the colonies were counted after 3 days of growth at 30°C. Both viable cell counts [colony forming units (CFU) per mL] and cell densities (OD_600_) were measured periodically over a total time of 96 h.

*E. coli* DH5α was used for cloning and production of the pUC18-based plasmids i.e., upprm-pUC and upsmo-pUC (for their description see below). DH5α cells were grown on LB at 37°C with shaking at 150 rpm. When appropriate, ampicillin was added to LB at a final concentration of 50 μg/mL.

### Whole cell experiments

*Rhodococcus* sp. strain BCP1 cells to be tested in the experiments for alcohol production and oxygen uptake activity were developed as follows. Five 119-mL bottles containing 40 mL of MM were prepared for each growth substrate. The cultures were grown up to a final OD_600_ ~ 1.0. After centrifugation, the cells were washed twice with 25 mM phosphate buffer (pH 7.2) and resuspended in 5 mL of the same buffer (herein concentrated cell suspension). Accumulation of 1- and 2-butanol was measured using 1-propanol or 2-propanol as inhibitors of butanol consumption, while the accumulation of 1- and 2-propanol was measured using 1-butanol and 2-butanol as inhibitors. The reaction mixture consisted of the substrate (*n*-butane or propane), competitor (5 mM), and O_2_-saturated phosphate buffer up to a total volume of 2 mL, inside 13.9 mL sealed serum vials. After 30 min equilibration of the reaction mixture at 30°C, the assay was initiated by the addition of the concentrated cell suspension (500 μL; around 200 mg wet mass of cells) into each sample vial. In the negative control samples, no inoculum was added. The vials were shaken at 150 rpm during the reactions. For inactivation assays, the concentrated cell suspension was added to the reaction mixture after being incubated with 5% (vol/gas-phase vol) acetylene for 30 min at 30°C with constant shaking. Liquid samples (4 μL) were removed after 15, 30, 45, and 60 min and the production of the short-chain aliphatic alcohols was determined by a gas chromatograph (GC, HP 5890) equipped with a flame ionization detector and a Supelcowax-10 column (30 m with 0.53 mm inner diameter). The GC was run at column temperature of 45°C for 5 min, then up to 200°C at 40°C/min, and with a detector temperature of 300°C. Helium was used as the carrier gas at a flow rate of 15 mL/min. Identities of products were determined by comparison of retention times and peak shapes to those of authentic compounds.

O_2_ consumption of BCP1 cells induced by the addition of various compounds was measured with a Clark-type O_2_ electrode inserted into a 1.9 mL chamber sealed with a capillary inlet through which additions were made. After BCP1 cells were grown in each condition under investigation, they were washed twice with 25 mM phosphate buffer (pH 7.2), suspended in the same buffer and shaken at 30°C for 5 h to decrease the endogenous respiration. Before being introduced into the electrode chamber, the biomass was diluted with air-saturated buffer to a final OD_600_ = 0.4. Cell suspensions were exposed to gases inside 13.9 mL sealed bottles, while the liquid substrates were introduced into the electrode chamber by direct addition. The reactions were carried out at Room Temperature (RT, 20–23°C) and stirred with a magnetic bar. In the inhibitory assay, an aliquot of the cell suspension from each condition of growth was incubated with acetylene (5% v/v) for 30 min before being introduced into the electrode chamber and being exposed to the substrates. The O_2_ consumed, expressed in nmols, was normalized to mg of proteins that was determined through method of Lowry (Lowry et al., 1951) with bovine serum albumine (BSA) as a standard. Experiments were conducted in triplicate.

### Nucleotide sequence analysis

RAST server was used to analyze the draft genome of *Rhodoccocus* sp. strain BCP1 that has been recently sequenced and published (Cappelletti et al., 2013). Sequence similarity searches were performed using BLAST programs (http://www.ncbi.nlm.nih.gov/blast/blast.cgi) (Altschul et al., 1997) together with the conserved domain database (http://www.ncbi.nlm.nih.gov/cdd/), whereas multiple sequence alignments were performed with ClustalW software (http://www.ebi.ac.uk/clustalw/). Phylogenetic trees were created using Geneiuos Tree Builder tool in Geneious Pro 4.7.6 software with the following parameters: genetic distance model, Juke-Cantor; tree-building method, neighbor joining; *Pseudonocardia tetrahydrofuranoxydans* K1 *thmA* and *Xanthobacter autotrophicus* Py2 *xamoA* were used as outgroup for *prmA* and *smoA*, respectively.

### Primer extension, reverse transcription (RT-) PCR and quantitative real time PCR (qRT-PCR) analysis

Total RNA was extracted from 100 mL of *Rhodococcus* sp. strain BCP1 culture grown at 30°C on MM enriched (MMenr) with yeast extract, casamino acids, peptone (0.5 g/L each), and 1% (wt/vol) succinate, up to a final OD_600_ = 0.6–0.7. After cell washing in phosphate buffer (10 mM; pH 7.2), each culture was suspended in 30 mL of MM. Cell cultures were incubated for 4 h in MM at 30°C in the presence of variable substrates. For the analysis of the effect of alternative carbon sources on *prmA* and *smoA* expression, BCP1 cells were grown on MMenr medium supplied with either glucose or succinate (1% w/v each), or on LB. After cell washing, propane or *n*-butane (150 μM) was introduced into sealed bottles with BCP1 cells in 30 mL of MM (for cells grown on MMenr) or LB (for cells grown on LB). In addition to gaseous *n*-alkanes, succinate or glucose was added to MM-resuspended cells at a final concentration of 1% (w/v). After incubation with the substrates, 1/10 volume of ice-cold phenol–ethanol solution was added to each bottle to stop the RNA degradation. The cells were precipitated and stored at −80°C for later use. RNA extraction protocol was performed as previously described by Cappelletti et al. (2011). The total RNA was treated twice with 5 U of RNase-free DNase (Qiagen; 30 min at 30°C) and purified with RNeasy kit (Qiagen).

For the cDNA synthesis, 500 ng amount of total RNA was reverse-transcribed with 0.25 μg of random hexamers (Invitrogen) in 10-μL reaction mixtures. After denaturation for 3 min at 94°C and annealing for 5 min at 37°C, 1 U of avian myeloblastosis virus (AMV) reverse transcriptase (Promega) and 50 U of the RNase inhibitor RNasin (Promega) were added and reverse transcription was performed at 42°C for 1.5 h. In order to study the co-transcription of the genes composing each cluster through RT-PCR assays, 1/10 of the resultant cDNA was amplified using the sets of primers listed in Table S1 and represented in Figure S1. After an initial incubation at 94°C for 2 min, 25 cycles of the following temperature program were used: 94°C for 30 s, 60°C for 30 s, and 72°C for 30 s. In order to exclude DNA contamination, negative controls were performed by omitting the reverse transcriptase in RT-PCR experiments, which were conducted with the same temperature program and the same primer sets for 30 cycles of amplification.

In order to assess the transcriptional level of *prmA* and *smoA* genes induced by the growth on *n*-alkanes added alone or in the presence of alternative carbon sources, Reverse Transcriptase-quantitative PCR (RT-qPCR) experiments were performed. For this purpose, the reverse-transcribed samples were amplified using the CFX96 Real-Time System (Bio-Rad). Each 20-μl qPCR volume contained 2 μL of the reverse-transcribed RNA samples, 10 μL of SsoAdvanced universal SYBR Green supermix (Biorad), and 250 nM of each primer (forward and reverse, listed in Table S1). Thermocycling conditions were as follows: 30 s at 95°C, followed by 30 cycles of 5 s at 95°C and 5 s at 60°C. Expression of the housekeeping gene, 16S rDNA, was used as the reference gene to normalize tested genes in *R*. sp. strain BCP1. The relative fold change in mRNA quantity was calculated for the gene of interest in each sample using the ΔΔ*Ct* method followed by Student's one-sample *t*-test to evaluate whether the gene targets were differentially regulated. Data are expressed as mean ± standard deviation derived from at least three independent experiments. Differences were considered significant at *P* < 0.05. For each RNA preparation, at least three independent real-time PCR experiments were conducted.

The primer extension assays were performed on RNA isolated from BCP1 cells that were exposed for 4 h to butane and succinate as previously described (Roncarati et al., 2007). The primers used to map the transcriptional start sites of *prmA* and *smoA* in primer extension assays are reported in Table S1. Primer extension products were analyzed on a 6% polyacrylamide gel next to a sequence ladder generated using the same primers and pUC18-based plasmids containing *prmA* or *smoA* and their 250-bp upstream regions (upprm-pUC or upsmo-pUC, respectively). Sequencing reactions were performed with the T7 sequencing kit (USB) as previously described (Cappelletti et al., 2011).

### Protein sample preparation and two-dimensional gel electrophoresis (2-DE) procedure

BCP1 cells were grown in MM supplied with the carbon source under analysis to the exponential phase (OD_600_ = 0.6–0.7). The biomass grown on each substrate was resuspended in 5 mL lysis buffer [8 M urea, 4% 3-(3-cholamidopropyl)-dimethylammonio-1-propanesulfonate (CHAPS), 2% IPG buffer in 10 mM Tris, pH 8.5, in the presence of 1 mM phenylmethylsulfonyl fluoride (PMSF)]. The suspension was aliquoted into 2 mL-tubes containing 0.5 mL of nitric acid pre-washed quartz beads (0.2–0.8 mm diameter, MERCK KGaA, Germany). The cells were disrupted using a Precellys®24 (*Bertin* Technologies) bead beater for five cycles of 30 s, speed 6000 m/s. Unbroken cells and cell debris were removed by low speed centrifugation (14,000 rpm, 10 min, 4°C) and the supernatants were cleared by ultracentrifugation (32,500 rpm, 1.5 h, 4°C). The cell-free protein extracts were either stored at −80°C or used immediately for proteomic studies.

The samples to be used for two-dimensional gel electrophoresis (2-DE) were concentrated, desalted and separated from low-molecular weight inhibitors by filtration through a 10-kDa filter (centrifugal filters with 10 kDa cut off, Millipore). The liquid remaining (retentate) in the reservoir was treated with RNase and DNase I (0.1 mg/mL each) for 30 min before being subjected to two additional filtration steps. In the end, around 50 μL of purified cell extract was transferred to a new tube and the amount of total soluble protein content was estimated by the Bradford assay (Bradford, 1976) that did show compatibility with the small concentration of urea still present in the sample. A volume of sample corresponding to 300 μg of extracted protein was solubilized in urea rehydration solution [8 M urea, 2% CHAPS, 2% IPG Buffer, 0.002% bromophenol blue, 25 mM dithiothreitol (DTT)] for 30 min at RT and then applied to Immobiline DryStrip (13 cm, non-linear pH 4–7) (GE Healthcare) for isoelectric focusing (IEF). IEF in the IPG strips was carried out for a total of 29.3 kVh at 20°C under mineral oil using ETTAN IPGphor (Amersham Biosciences). After IEF, the strips were equilibrated for 30 min at RT in equilibration solution (40% glycerol, 50 mM Tris pH 8.8, 8 M urea, 2% SDS) containing 2% DTT and then in equilibration solution containing 2.5% iodoacetamide. Proteins were separated by 12% sodium dodecyl sulfate polyacrylamide gel electrophoresis (SDS-PAGE) in the second dimension using a PROTEAN II xi 2-D Cell electrophoresis system (gel size 16 × 20 cm) (BioRad). For protein spot visualization, 2-D gels were stained with Coomassie brilliant blue staining.

### LC/MS/MS analyses and protein identification

Protein spots were excised from Coomassie brilliant blue-stained 2-D gels, washed and prepared for LC/MS/MS analyses as described by Tremaroli et al. (2011). Tandem mass spectra (LC/MS/MS) were obtained on an Agilent 1100 Series LC/MSD ion Trap XCT Plus as previously reported (Tremaroli et al., 2011). Proteins were identified using the MASCOT search engine (www.matrixscience.com) and a BCP1 protein database that was built up in conjunction with the genome-sequencing study (Cappelletti et al., 2013). Theoretical molecular masses and isoelectric points of the proteins of interest were calculated using EXPASY tools (Bjellqvist et al., 1993).

## Results

### BCP1 growth on short-chain *n*-alkanes and on putative metabolic intermediates

It has previously been reported that *Rhodococcus* sp. strain BCP1 has the capacity to grow on *n*-alkanes (Frascari et al., 2006; Cappelletti et al., 2011). Here, the analysis of BCP1 growth has been extended to C_1_–C_7_ *n*-alkanes along with potential intermediate metabolites of gaseous *n*-alkanes (1- and 2-propanol/butanol, propionic and butyric aldehydes, propionic and butyric acids, acetone, and butanone) (see Table 1). Except for C_1_, BCP1 cells grew on the tested short-chain *n*-alkanes and showed the lowest Generation Times (G) with C_3_–C_5_ *n*-alkanes (2.7–2.8 h). Growth of BCP1 was also seen on all the assayed potential intermediate metabolites such as alcohols and fatty acids. Better growth was observed using the propane and *n*-butane oxidation products containing a hydroxyl group (–OH) at the terminal position, 1-propanol and 1-butanol, than compared to the sub-terminal oxidized alcohols, 2-propanol, and 2-butanol (Table 2). Higher final OD_600_-values were reached by BCP1 cultures growing on butyrate and propionate than compared to butanone and acetone; these latter compounds being the oxidation products of 2-butanol and 2-propanol, respectively. Nevertheless, the substrate range experiments indicate the presence of enzymes transforming putative metabolic intermediates of both sub-terminal and terminal oxidation pathways in BCP1 metabolism of gaseous *n*-alkanes.

**Table 1:** **Growth on short-chain *n*-alkanes by *R*. sp. strain BCP1^a^**.

**Substrate**	**Growth (change in OD_600_ after 96 h)**	**G (h)^b^**
Ethane	0.87±0.02	3.6±0.4
Propane	0.87±0.03	2.7±0.5
Butane	0.90±0.06	2.8±0.2
Pentane	0.84±0.10	2.7±0.4
Hexane	0.82±0.11	3.5±0.4
Heptane	0.73±0.05	3.7±0.4
1-Propanol	0.79±0.07	2.5±0.5
2-Propanol	0.56±0.01	3.6±0.4
1-Butanol	0.85±0.04	2.9±0.3
2-Butanol	0.65±0.01	3.7±0.4
Propionic aldehyde	0.23±0.02	2.8±0.6
Butyric aldehyde	0.28±0.06	2.9±0.5
Propionate	0.50±0.03	2.7±0.6
Butyrate	0.49±0.01	2.7±0.7
Acetone	0.25±0.05	3.7±0.3
2-Butanone	0.31±0.04	5.2±0.4
Succinate	0.36±0.04	3.8±0.3

a *Average values ± standard deviations*.

b *Generation time*.

**Table 2:** **Ability of *R*. sp. strain BCP1 to oxidize *n*-alkanes (*n*-butane and *n*-hexane) and potential intermediates of *n*-butane metabolism after batch growth on succinate, *n*-butane, *n*-hexane, 1-butanol, and butyric aldehyde. O_2_ uptake rates are expressed as nmol oxygen consumed min^−1^ (mg protein)^−1^. ND, not determined ^a^**.

**Assay substrate**	**Growth substrate**				
	**Succinate**	***n*-Butane**	***n*-Hexane**	**1-Butanol**	**Butyric aldehyde**
*n*-Butane	13±9	91±6	89±21	0	2.9±1.2
*n*-Hexane	6±3	74±8	103±26	0	7.3±2.3
1-Butanol	46±16	84±11	117±25	169±25	39±7
2-Butanol	22±16	90±10	90±10	112±11	34±6
Butyric aldehyde	48±10	95±12	92±3	86±10	66±11
Butyric acid	0	7±2	5±2	ND	ND
*n*-Butane (acetylene)	11±2	8±4	11±8	ND	ND

a *Average values ± standard deviation*.

In order to test the inducible nature of the enzyme systems involved in the short-chain *n*-alkanes metabolism, the O_2_ consumption activities of BCP1 cells grown on *n*-butane (gaseous alkane), *n*-hexane (liquid alkane), 1-butanol, butyric aldehyde (both C_4_ metabolic intermediates), and succinate (control), were determined by polarography (Table 2). The stimulation of cellular O_2_ uptake was measured upon addition of the same *n*-alkanes or the putative metabolic intermediates of *n*-butane metabolism. Results show that the *n*-alkane oxidative system was not induced in cells grown on *n*-butane metabolic intermediates (1-butanol, butyric aldehyde) and was only slightly induced (around 10–15% of the values obtained from cultures grown on *n*-alkanes) in cells grown on succinate. The addition of *n*-hexane stimulated O_2_ consumption in *n*-butane-grown cells and the same effect was observed with the addition of *n*-butane to *n*-hexane-grown cell suspensions. These results indicate that the monooxygenases (MO) induced by growth on either *n*-butane or *n*-hexane have a substrate range including both these short-chain *n*-alkanes. Cells pre-incubated with acetylene, a known MO inactivator, decreased the O_2_ consumption rates induced by *n*-alkanes by 70–75%. The constitutive metabolism of 1-butanol and butyric aldehyde was demonstrated by the O_2_ uptake observed with succinate-grown cells after the addition of these substrates to the cell suspensions. An increase in the level of O_2_ consumption induced by *n*-butane metabolic intermediates was detected with BCP1 cells grown on *n*-butane but also on *n*-hexane. These results suggest that the enzymatic systems acting downstream of the initial *n*-hexane oxidation production have a group of substrates which includes *n*-butane metabolic intermediates. The O_2_ consumption activity of 1-butanol-grown cells was substantially stimulated by the addition of either 1-butanol or 2-butanol and only slightly stimulated by the addition of butyric aldehyde (Table 2). The absence of butyric acid oxidation by *n*-butane- and *n*-hexane-grown cells might be due to the lack of induction of an uptake system for this substrate during the growth on *n*-alkanes (Salanitro and Wegener, 1971).

### Gas-chromatographic (GC) analyses of gaseous *n*-alkanes oxidation

GC analyses were performed to define the oxidation products of short-chain *n*-alkane metabolism by BCP1 resting cells pre-grown on either *n*-butane or propane. The accumulation of the C_4_ and C_3_ oxidation products in the growth medium was obtained by adding excess amounts of their structural analogs. When 1-propanol was supplied in addition to *n*-butane to the suspension of *n*-butane-grown BCP1 cells, 1-butanol accumulated and was already detected as a product of *n*-butane oxidation after 15 min of incubation (Figure S2). The amount of 1-butanol produced was not stoichiometrically equivalent to the amount of *n*-butane consumed; this was possibly due to only partial inhibition exerted by 1-propanol on the downstream oxidation steps. To determine whether *n*-butane undergoes subterminal oxidation in BCP1 cells, 2-propanol was supplied as competitor in addition to *n*-butane. As a result, no 2-butanol production was detected (Figure S2). However, when propane-grown cells were tested in the presence of propane and excess amount of either 1-butanol or 2-butanol, peaks corresponding to 1-propanol and 2-propanol were observed, respectively (Figure S2). The accumulation of the oxidation products was completely inhibited when *n*-butane- or propane-grown BCP1 cells were pre-treated with acetylene, a well-known monooxygenase inactivator (data not shown). The production of 1-butanol from *n*-butane and 1-propanol from propane indicates that the metabolism of short-chain *n*-alkanes undergoes terminal oxidation process in BCP1. However, the production of 2-propanol suggests that subterminal oxidation reaction can also occur.

### Analyses of two *sdimo* gene clusters in *Rhodococcus* sp. strain BCP1

The analysis of the genome of BCP1 revealed two gene clusters coding for SDIMOs. Each cluster included four genes encoding the following monooxygenase components: two subunits of the hydroxylase, an effector (or coupling) protein and a reductase. The single genes were arranged differently in the two clusters and their nucleotide sequences shared 48–55% identity. Based on similarity analysis with SDIMO sequences present in the database and phylogenetic analysis of genes coding for the SDIMO alpha subunits (Figures S3, S4 in Supplementary Material), the two gene clusters were named *prmABCD* and *smoABCD*.

**Figure 1:**
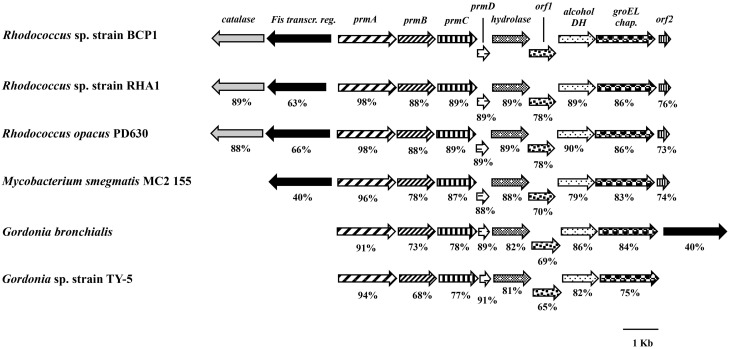
**Organization of the *prmABCD* cluster and the flanking regions from *R*. sp. strain BCP1 and comparisons with equivalent regions from other bacteria (only homologous genes are represented)**. The locus tags of the genomic regions taken into consideration are as follows: KDE11336–KDE11346, *R*. sp. strain BCP1*;* RHA1_ro00439–RHA1_ro00449, *R*. sp. strain RHA1; OPAG_05101–OPAG_05111, *R. opacus* PD630; MSMEG_1970–MSMEG_1979, *Mycobacterium smegmatis* MC2 155*;* Gbro_3551–Gbro_3559, *G. bronchialis*; BAD03956–BAD03963, *G*. sp. strain TY-5. Genes are represented by arrows and homologous genes are represented by the same pattern. Full names for abbreviated genes or products are listed as follows: *prmA*, oxygenase large subunit; *prmB*, reductase; *prmC*, oxygenase small subunit; *prmD*, coupling protein; groEL chap., chaperonine beloning to GRoEL family; DH, dehydrogenase; Fis transcr. reg., transcriptional regulator belonging to Fis family; *orf1*, metal-sulfur cluster biosynthetic enzyme; *orf2*, antibiotic biosynthesis monooxygenase. Numbers underneath the arrows indicate the percentage of amino acid sequence identity between each encoded gene product and the equivalent product from *R*. sp. strain BCP1.

The BCP1 *prmABCD* gene covers a 4,255 bp chromosomal DNA region (Gene Accession number NZ_CM002177) comprising of the following four consecutive ORFs homolog to the propane monooxygenase (*prm*) gene cluster components: *prmA* (oxygenase large subunit), *prmB* (reductase), *prmC* (oxygenase small subunit), *prmD* (coupling protein). Considering the blast hits described in literature, the BCP1 *prmABCD* cluster has the same operon arrangement as the homologous regions from *R*. sp. strain RHA1 (*prmABCD* operon), *Mycobacterium* (*M*.) *godii* (*mimABCD* operon) and *Gordonia* sp. strain TY-5 (*prmABCD* operon) in addition to showing high similarity (80–89% nucleotide identity) to these regions. It also shares 69 and 74% similarity with the *Pseudonocardia* sp. strain TY-7 *prm1ABCD* and *prm2ABCD* operons, respectively. Intergenic distances between the stop codons and the start codons were 98 bp for *prmA* and *prmB* and 50 bp for *prmB* and *prmC*, whereas the 3′ terminal nucleotide of *prmC* and the 5′ terminal nucleotide of *prmD* overlapped by 4 bp. Putative Shine-Dalgarno-like ribosome binding site (RBS) sequences (AGGAGG, AGGAG, AGAAG, AAGGAG) were found upstream of *prmA, prmB, prmC*, and *prmD*. Regarding the *prmABCD* flanking regions, the relative position and transcription direction were maintained for some of these genes as compared to homologous regions belonging to other reference strains which have their complete genomes sequenced, i.e., *Rhodococcus* sp. strain RHA1, *R. opacus* PD630, *M*. *smegmatis, Gordonia bronchialis* (Figure 1). The conserved genes are located downstream of the *prmABCD* cluster and code for a metal-dependent hydrolase, a metal-sulfur cluster biosynthetic enzyme, an alcohol dehydrogenase, and a GroEL chaperon. In *M. smegmatis* RHA1, PD630, and BCP1, additional conserved genes code for a Fis family transcriptional regulator and an antibiotic biosynthesis monooxygenase. A gene coding for a catalase is maintained in the three *Rhodoccocus* spp. (Figure 1). In *Rhodococcus* and *Mycobacterium*, the Fis family transcriptional regulator gene is located upstream of *prmA* and transcribed in the opposite direction to *prmABCD*. In *Gordonia*, the homolog of BCP1 Fis regulator gene, is positioned downstream of the conserved region comprising the *prm* gene cluster, and is transcribed in the same direction (Figure 1).

**Figure 2:**
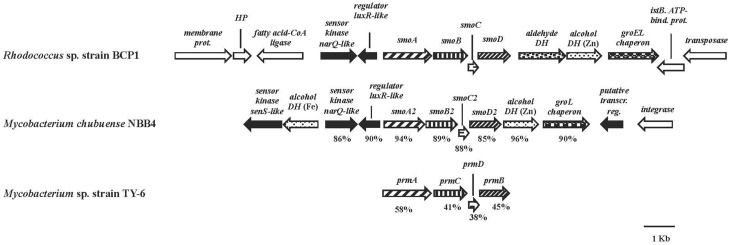
**Organization of the *smoABCD* cluster and the flanking regions from *R*. sp. strain BCP1 and comparisons with equivalent regions from other two *Mycobacterium* strains**. *smoA2B2C2D*2 and *prmACDB* are the only gene cluster functionally described among the best BLAST hits of BCP1 *smo* genes (Kotani et al., 2006; Coleman et al., 2011). The locus tags of the genomic regions taken into consideration are as follows: KDE09885–KDE09898, *R*. sp. strain BCP1*;* Mycch_5399–Mycch_5388, *M. chubuense* NBB4; AB250938, *M*. sp. TY-6. Genes are represented by arrows. Homologous genes are represented by the same pattern except for the white arrows that represent genes without homology with other genes displayed in the figure and the black arrows that represent regulatory genes. Full names for abbreviated genes or products are listed as follows: *smoA/smoA2*, monooxygenase alpha subunit; *smoB/smoB2*, monooxygenase beta subunit; *smoC/smoC2*, coupling protein; *smoD/smoD2*, reductase; *prmA*, propane monooxygenase hydroxylase large subunit; *prmC*, propane monooxygenase hydroxylase small subunit; *prmD*, propane monooxygenase coupling protein; *prmB*, propane monooxygenase reductase. Full names for abbreviations are: HP, hypothetical protein; DH, dehydrogenase; bind., binding; prot., protein; transcr. reg., transcriptional regulator. Numbers underneath the arrows indicate the percentage of amino acid sequence identity between each encoded gene product and the equivalent product from *R*. sp. strain BCP1.

The *smoABCD* gene cluster in BCP1 is found on the 103,129 bp plasmid pBMC2 (Gene Accession number NZ_CM002179) and covers a 3,987 bp region (Figure 2). As inferred from both RAST annotation and BLAST analyses, the single genes code for the monooxygenase alpha subunit (SmoA), the monooxygenase beta subunit (SmoB), the coupling protein (SmoC) and the reductase (SmoD). The nucleotide sequence of *smoABCD* gene cluster exhibited overall identities of 85 and 81% with homologous regions from *M. chubuense* NBB4 and *M. marinum*, respectively. Notably, a few blast hits matched the amino acid sequence of SmoA with an identity score higher than 80% which underlines the scarcity of homologs to this SDIMO in the database. The intergenic distance between the stop codon of *smoC* and the start codon of *smoD* is 11 bp. *smoA/smoB* and *smoB/smoC* genes overlapping regions are of 1 and 4 bp, respectively. A putative Shine-Dalgarno-like RBS sequence (GGGAG) was found 7 bp upstream of *smoA*. Located upstream of *smoABCD*, there is a gene for long-chain fatty acid CoA ligase as well as two genes coding for two-component system regulators. The latter two genes are also maintained in the homologous region of *M. chubuense* NBB4 (Figure 2). Furthermore, two genes encoding a zinc-containing alcohol dehydrogenase (DH) and a cpn60-type chaperonin are located downstream of both BCP1 *smoABCD* and NBB4 *smoABCD* regions; although in BCP1, an additional gene encoding an aldehyde DH is also present (Figure 2).

### Transcriptional analysis of *prm* and *smo* gene clusters in BCP1 cells exposed to *n*-alkanes

**Figure 3:**
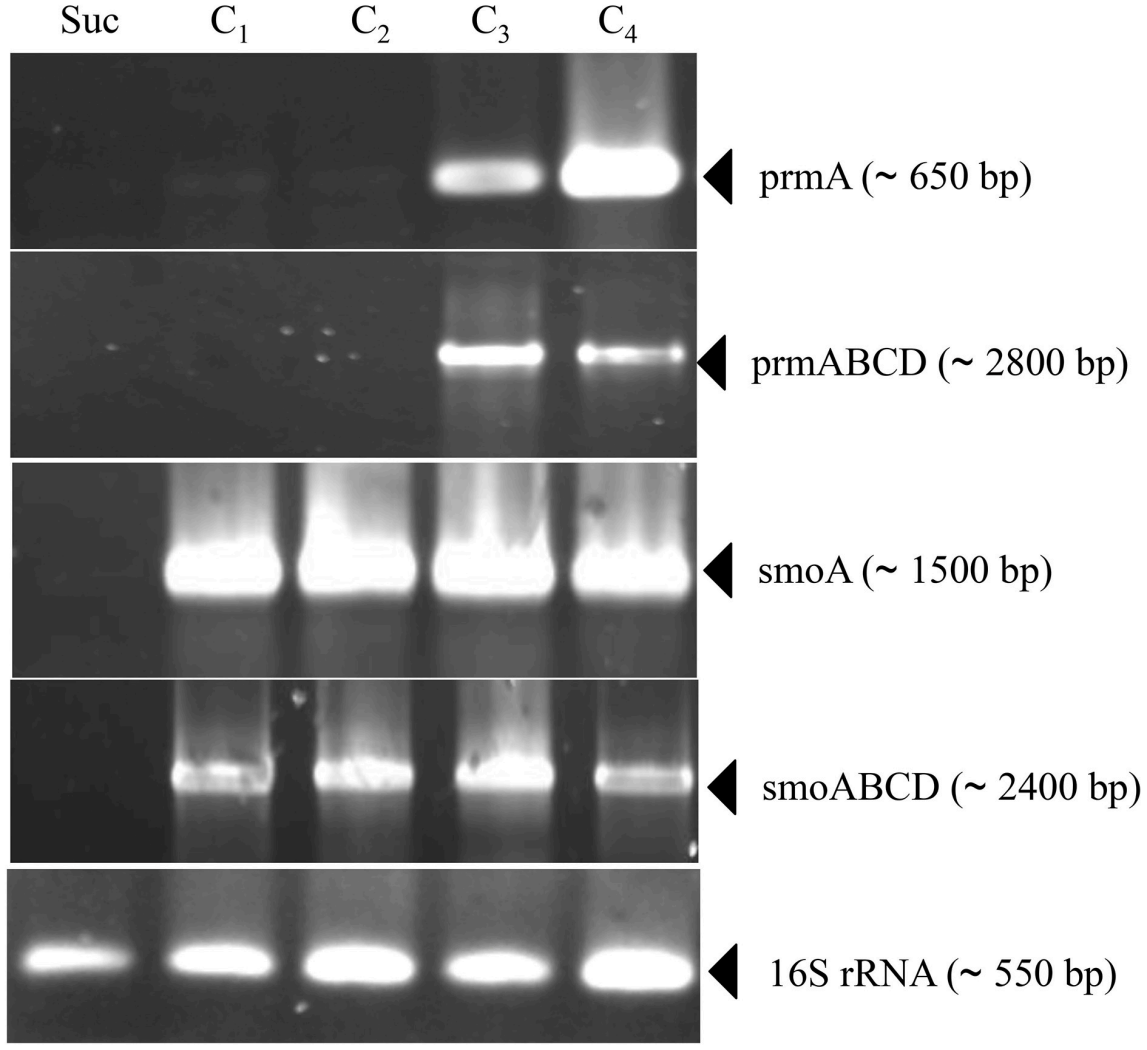
**RT-PCR analysis of *prmA, smoA, prmABCD* and *smoABCD* from *R*. sp. strain BCP1**. mRNA was prepared from cells of BCP1 which have been exposed for 4 h to succinate (Suc), methane (C_1_), ethane (C_2_), propane (C_3_), *n*-butane (C_4_). The different sets of primers that have been used to amplify a RT-PCR product for each gene (*prmA* and *smoA*) and for each gene cluster (*prmABCD* and *smoABCD*) are reported in Table S1 and represented in Figure S1. The size of each PCR product is indicated between brackets.

Information on the transcriptional inducibility of the two *sdimo* gene clusters and their transcriptional organization was obtained through RT-PCR experiments. To this end, the alpha subunit coding genes (*prmA* and *smoA*) as well as the entire gene clusters (*prmABCD* and *smoABCD*) were targeted in RT-PCR using RNA from BCP1 cells exposed for 4 h to gaseous *n*-alkanes (C_1_–C_4_) and succinate. As a result, DNA fragments with expected sizes were obtained for *prmA* gene and *prmABCD* operon using the total RNA from BCP1 cells grown on propane and *n*-butane (Figure 3). No transcription products were revealed from BCP1 cells exposed to methane or grown on ethane and succinate. RT-PCR products for *smoA* and *smoABCD* were detected using the RNA of BCP1 cells exposed to all the tested *n*-alkanes (C_1_–C_4_), but not to succinate (Figure 3). No amplification products were obtained when reverse transcriptase was omitted from the reaction mixtures (data not shown). These results qualitatively indicate that a different range of substrates induces the expression of the two *sdimo* gene clusters and that they are transcribed as a polycistronic unit.

**Figure 4:**
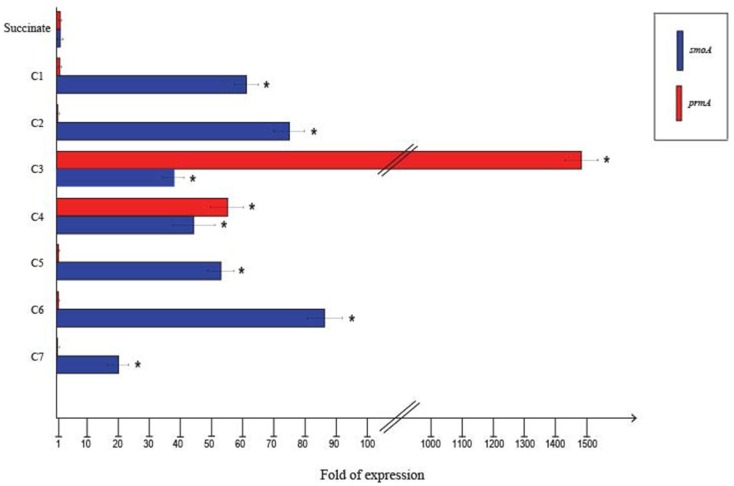
**Fold changes in *prmA* and *smoA* genes expression induced in *R*. sp. strain BCP1 cells incubated with C_1_–C_7_*n*-alkanes assayed by RT-qPCR**. Fold increases were calculated relative to the level of BCP1 cells incubated with sodium succinate (control). Asterisks denote that the expression values obtained with short-chain *n*-alkanes-grown cells are statistically different from those with succinate-grown cells based upon Student's test (*P* < 0.05). Mean values of three independent experiments with standard deviations are presented.

To quantify the differential expression of *smoA* and *prmA* genes induced by different short-chain *n*-alkanes, RT-qPCR experiments were carried out using RNAs extracted from BCP1 cells exposed for 4 h to short-chain *n*-alkanes (C_1_–C_7_) and succinate. Before using the RNA preparations for cDNA synthesis, the absence of genomic DNA was evaluated for each sample by performing RT-PCR experiments without the addition of the reverse transcriptase (see Materials and Methods). As shown in Figure 4, the mRNA level of *prmA* was around 1500- and 50-fold higher when grown on propane and *n*-butane, respectively, vs. succinate as the sole carbon source. Conversely, the *prmA* transcriptional level induced by the growth on C_1_, C_2_, C_5_–C_7_ *n*-alkanes was not significantly higher than that induced by succinate (<5-fold). The expression levels of *smoA* in response to the presence of *n*-alkanes were 20- to 85-fold higher compared to succinate (Figure 4).

**Figure 5:**
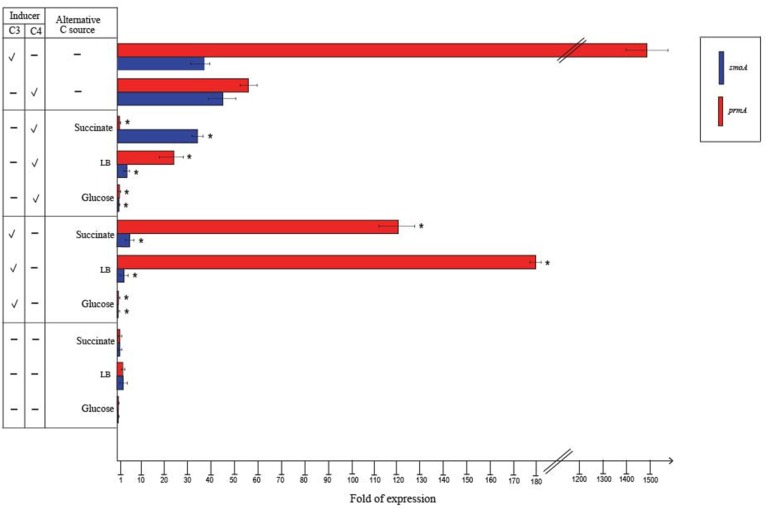
**Fold changes in *prmA* and *smoA* genes expression induced in *R*. sp. strain BCP1 cells incubated with propane (C_3_) or *n*-butane (C_4_) (inducers) in the presence of succinate, glucose or LB (alternative carbon source) assayed by RT-qPCR**. The symbols stand for: –, absence; V, presence. Fold increases were calculated relative to the level of BCP1 cells incubated with sodium succinate (control). Asterisks denote that the expression values with alternative carbon sources + inducers are statistically different from those with the only inducers based upon Student's *t*-test (*P* < 0.05). Mean values of three independent experiments with standard deviations are presented.

RT-qPCR experiments were also carried out to evaluate the effect of alternative carbon sources on the expression of *prmA* and *smoA* (Figure 5). In the presence of glucose or when grown on LB, the expression level of both the *sdimo* genes was similar to that observed on succinate. When BCP1 cells were exposed to either propane or *n*-butane in the presence of a TCA cycle organic acid (succinate), a simple sugar (glucose) or during their growth on complete medium LB, a decrease in the transcriptional induction of both the genes was observed. In particular, when glucose was added in the presence of either propane or *n*-butane the expression of both *prmA* and *smoA* was completely repressed (Figure 5). The growth of BCP1 on LB in the presence of propane or *n*-butane repressed the *smoA* expression. Growth on LB with propane or *n*-butane also reduced the *prmA* expression by 8- and 2.5-fold as compared to growth on minimal medium supplied with only propane and only *n*-butane, respectively. The inclusion of succinate completely repressed the *prmA* expression in the presence of *n*-butane. In the presence of propane, succinate addition reduced *prmA* and *smoA* transcription by 13- and 8-fold, respectively. Therefore, a carbon catabolite repression mechanism affects the expression of the two *sdimo* genes depending on the type of inducer (propane or *n*-butane) and alternative carbon source supplied.

### Analysis of the promoters of *prmABCD* and *smoABCD* operons

**Figure 6:**
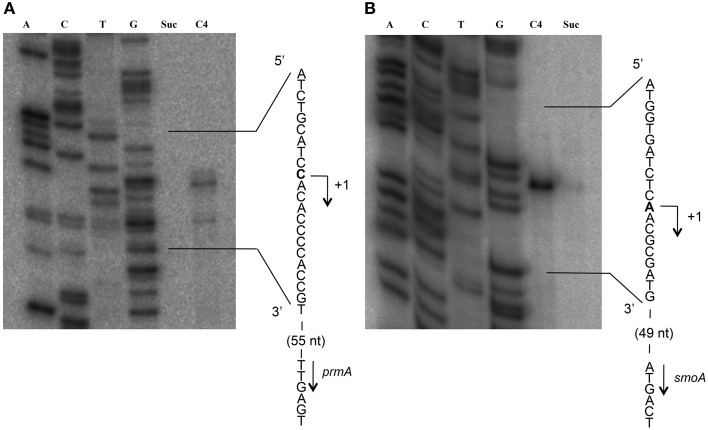
**Identification of the transcriptional start sites of *prmA* (gel A) and *smoA* (gel B) genes in *R*. sp. strain BCP1**. The polyacrylamide gels show the primer extension products obtained from the total RNA of BCP1 cells exposed to succinate (Suc) or *n*-butane (C_4_) using the primer PEprm7 (gel **A**) or PEsmo1 (gel **B**). Lanes A, T, C, and G in the two gels show the products of the sequencing reactions using as template the plasmid containing the upstream region of *prmA* (gel **A**) or *smoA* (gel **B**) (See Material and Methods). The deduced transcriptional start sites for the two genes are shown in bold, and the direction of transcription is indicated by an arrow on each sequence. The distances between each start site and the corresponding start codon are also indicated in parentheses.

To define the transcriptional start sites (TSS) of the two *sdimo* operons, primer extension assays were conducted using BCP1 cells exposed to *n*-butane and succinate as control (Figure 6). Two different radioactively labeled oligonucleotides annealing at different positions in the 5′ region of each of the two alpha subunit coding genes were used: (A) PEprm7 and PEprm8, which mapped to nucleotides 50 and 24 downstream of the TTG codon of the *prmA* gene, respectively; (B) PEsmo1 and PEsmo2, which mapped to nucleotides 90 and 75 downstream of the ATG codon of the *smoA* gene, respectively (Table S1). The TSS determined were a cytosine 55 bp upstream of the *prmA* initiation codon (Figure 6A) and an adenine 49 bp upstream of the *smoA* initiation codon (Figure 6B).

**Figure 7:**
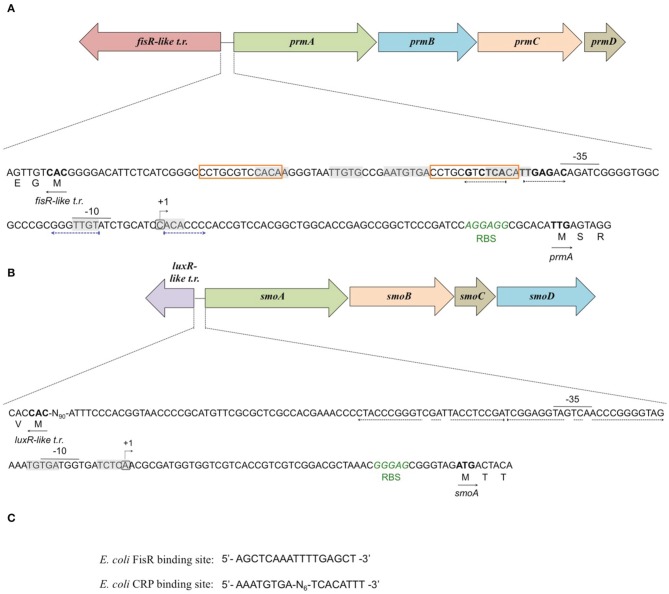
**Analysis of the promoters of *prmA* (A) and *smoA* (B) genes in *R*. sp. strain BCP1**. The transcriptional start sites detected by primer extension experiments for each *sdimo* are circled and indicated by a bent arrow. The putative −10 and −35 promoter regions as well as the ribosome-binding sites (RBS) are indicated below each sequence. The direct repeat sequences upstream of *prmA* gene are indicated in orange boxes **(A)**. The putative CRP (catabolite repression protein) binding sites are shaded in gray. The nucleotides forming the inverted repeats are highlighted with divergently oriented (black or blue) arrows. The nucleotides matching the Fis regulator high affinity binding site described in *E. coli* (shown in **C**) are in bold. The translational start codon of each gene is in bold with an arrow underneath indicating the direction of transcription/translation. *t.r*. stands for transcriptional regulator.**(C)** shows the DNA sequences binding the Fis regulator and the catabolite repression protein (CRP) in *E. coli*.

Putative −35 and −10 hexamer identification was based on the sequences of other *Rhodoccocus* promoters (Veselyì et al., 2007; Tomás-Gallardo et al., 2012) and motif features such as the distance between the hexamers and the TSS. The hypothetical −10 (TTGTAT) region is depicted upstream of *prmA*, separated by 7 bp from the TSS (Figure 7A, Figure S5). Considering the distance of 16–18 bp between the regulatory hexamers, a −35 region (CAGATC) was also proposed for *prmA* in Figure 7A. In Figure 7B, hypothetical −35 (TAGTCA) and −10 (GATGGT) regions are presented for *smoA* gene and are separated by 17 bp. Putative CRP (catabolite repression protein) binding sites were also identified in the promoter regions of both the *sdimo* gene clusters under analysis (Figure 7). The putative CRP binding sites that are immediately upstream of the −35 regions of *prmA* and those that partially overlap the −10 region and the TSS of *smoA* are conserved among homologous regions (Figure S5). These results support experimental data on the transcriptional repression of *prmA* and *smoA* observed when alternative carbon sources are added along with inducers (propane or *n*-butane) into growth medium. Additional conserved palindromic sequences were detected covering the putative −35 boxes of both the *sdimo* genes (Figure 7, Figure S5). Notably, the palindromic sequence upstream of *prmA* gene shows some similarities with the symmetrical consensus sequence identified by Shao et al. (2008) for being a high affinity binding site for Fis regulators in *E. coli* (Figure 7C). Regions with peculiar features included in the BCP1 *prmA* promoter region are two imperfect 13 bp-direct repeat sequences (orange boxes in Figure 7A) and a palindromic sequence overlapping the −10 region (divergently oriented blue arrows in Figure 7A). It is interesting to note that these special regions are not conserved among promoters of *prmA* homologous genes present in the database (Figure S5).

### Identification of short-chain *n*-alkane-induced proteins by two-dimensional electrophoretic analysis

**Figure 8:**
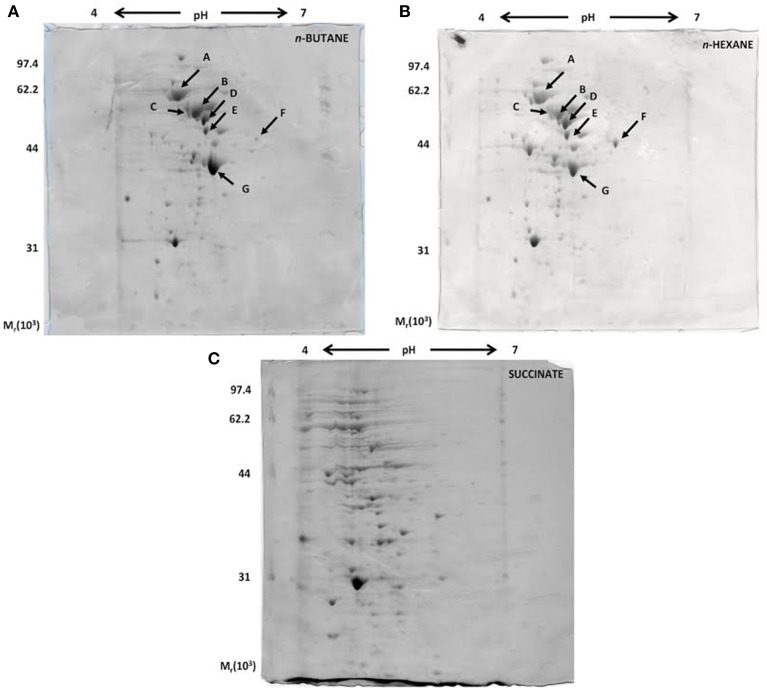
**Coomassie blue-stained 2-D SDS-PAGE gels representing the cytoplasmic fractions of *R.* sp. strain BCP1 cells grown on *n*-butane (A), *n*-hexane (B), and succinate (C)**. The seven protein spots indicated by arrows in **(A)** and **(B)**, which are missing in **(C)**, were further analyzed.

A proteomic approach was used to provide insights into the metabolic pathways of short-chain *n*-alkane catabolism in *Rhodococcus* sp. strain BCP1. Soluble protein extracts from cells grown with either *n*-butane (gaseous alkanes) or *n*-hexane (short-chain liquid alkane) as carbon source were compared by 2-D gel analysis. Soluble protein extract from cells grown on succinate was used as a reference. Initial data from a 2-D gel using a IPG strip from pH 3 to 10 revealed that most of the cytoplasmic proteins focused in regions corresponding to pH-values below 7 (data not shown). Consequently, IPG strips from pH 4 to 7 were used for further analysis. Representative examples of 2-D gels are shown in Figure 8. The protein patterns produced in *n*-butane- and *n*-hexane-grown cells were similar to each other, and less comparable to that obtained with succinate-grown cells.

Our present analysis has focused on the study of seven protein spots (A–G) that were induced by the growth on the two *n*-alkanes (Figure 8), but not on succinate. LC/MS/MS data collected on the protein content of each of the seven spots under analysis are summarized in Table S2. Interestingly, among the proteins identified, the aldehyde dehydrogenase (DH) KDE09894 and the alcohol DH KDE09895 are coded by consecutive genes located downstream of the *smoABCD* operon. Other alcohol and aldehyde DHs have been detected that are coded by genes with locations on the genome not related with that of monooxygenase genes described in this paper. The up-regulated proteins are involved in the terminal oxidation pathway of the *n*-alkanes, as well as in fatty acid metabolism, the glyoxylate bypass and protein folding.

## Discussion

In this work, the *Rhodococcus* sp. strain BCP1 growth on short-chain *n*-alkanes was investigated through the analysis of the following aspects, namely: (i) the rate of cell growth; (ii) the product(s) of the initial oxidation step(s), (iii) the induction of O_2_ consumption, (iv) the transcriptional induction of two gene clusters coding for SDIMOs, and, finally, (v) the protein pattern induced by short-chain *n*-alkanes.

**Figure 9:**
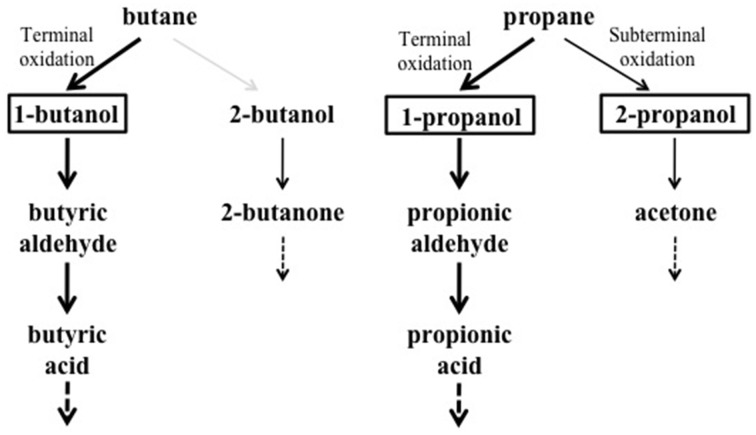
**Putative metabolic pathways of gaseous*n*-alkanes in *R*. sp. strain BCP1 based on the growth data and oxidation products described in this work**. The compounds that can be used as growth substrates by BCP1 are in bold. The detected metabolic intermediates are boxed. Thicker arrows indicate the predominance of the terminal oxidation pathways over the subterminal one suggested by BCP1 growth data (OD_600_ and generation time). The gray arrow indicates the lack of detection of 2-butanol among the butane oxidation products. The dashed arrows indicate that the intermediates are further metabolized.

Under the growth conditions examined here, the strain BCP1 has shown generation times ranging from 2.7 to 4.3 h, the fastest growth rates seen on C_3_–C_5_ *n*-alkanes (G of 2.6–2.7 h). BCP1 cells utilize metabolic intermediates of both terminal and sub-terminal oxidation pathways of gaseous *n*-alkanes, although shorter generation times and higher OD_600_-values were observed with 1-butanol and 1-propanol compared to 2-butanol and 2-propanol (Table 1). Further, the GC-analysis revealed that 1-butanol was a product of *n*-butane oxidation and both 1-propanol and 2-propanol were products of propane oxidation. These results suggest the predominance of the terminal oxidation pathway in the gaseous *n*-alkanes metabolism of BCP1 cells (Figure 9). The capacity of generating both terminal and sub-terminal oxidation products from propane has also been reported in *Rhodococcus rhodochrous* PNKb1 (Perry, 1980; Woods and Murrell, 1989; Ashraf et al., 1994), while *Gordonia* sp. strain TY5 was shown to oxidize C_3_ sub-terminally to yield 2-propanol (Kotani et al., 2003). In *M. vaccae* JOB5, propane is metabolized via either subterminal or terminal oxidation, while *n*-butane is metabolized via terminal oxidation only (Phillips and Perry, 1974).

Previous studies have shown that different types of Fe-MOs are induced during the growth of BCP1 on gaseous (propane, *n*-butane) and liquid (*n*-hexane) short chain *n*-alkanes (Frascari et al., 2006; Cappelletti et al., 2010, 2011). However, as shown in Table 2, quite similar O_2_ consumption rates were measured after the addition of either one of the two *n*-alkanes (*n*-butane or *n*-hexane) to *n*-butane- and *n*-hexane-grown cells. Apparently, these results indicate that monooxygenases with relaxed substrate specificity are involved in the oxidation of liquid and gaseous short chain *n*-alkanes.

Here, the expression of two gene clusters (*prmABCD* and *smoABCD*) detected in BCP1 genome and coding for two SDIMOs, has been analyzed. These clusters contain four genes each encoding the large and small subunits of the hydroxylase, the reductase and the coupling protein (Figures 1, 2). Interestingly, the organization of the two gene clusters is different and their similarity in terms of nucleotide sequences is low (around 50%). The *prmABCD* gene cluster has a chromosomal localization while *smoABCD* is located on pBMC2 endogenous plasmid (Cappelletti et al., 2013). This is in line with other studies reporting that large linear plasmids comprise biodegradative genes involved in catabolic functions such as oxygenases linked to hydrocarbon degradation (Masai et al., 1997; Saeki et al., 1999).

The transcription of BCP1 *prmABCD* and *smoABCD* genes is induced by the growth on *n*-alkanes and they are transcribed as polycistronic units. In particular, the expression of *prmA* was strongly induced by the growth on propane with a propane/succinate ratio close to the propane/pyruvate ratio described for *prmA* gene in *R. jostii* RHA1 (1480 vs. 2450, respectively) (Sharp et al., 2007). Here we show that BCP1 *prmA* expression is also induced by the growth on *n*-butane but not by the growth on methane, ethane, *n*-pentane, *n*-hexane, *n*-heptane (Figure 4). On the other hand, BCP1 *smo* gene cluster transcription was induced by all the *n*-alkanes under analysis in this study and not by succinate (Figure 4). Surprisingly, the inducing range of *smoA* comprises C_1_ that is not utilized by BCP1 for growth (Frascari et al., 2006). The homolog of *smoA* in *Mycobacterium chubuensis* NBB4 (Gene ID: Mycch_5395) was reported to be induced during the growth on acetate, ethene, and C_2_–C_4_ alkanes although no quantitative information on transcriptional level was provided (Coleman et al., 2011). In Table 3, the ability of BCP1 to grow on gaseous *n*-alkanes (propane, *n*-butane) is related to the expression of the two *sdimo* genes described in this work, while the growth on *n*-hexane is related to the expression of *alkB* gene reported in a previous study (Cappelletti et al., 2011).

**Table 3:** **Summary of the metabolic and genetic aspects concerning the *Rhodococcus* sp. strain BCP1 growth on short-chain *n*-alkanes or succinate**.

**Growth substrate**	**G (h)**	**Gene expression**			**Oxidation products**
		alkB^*^	***prm***	***smo***	
Propane	2.7 ± 0.5	-^#^	+++	+	1-Propanol
					2-Propanol
*n*-Butane	2.8 ± 0.2	−	+	+	1-Butanol
*n*-Hexane	3.5 ± 0.4	+	−	+	nd
Succinate	3.8 ± 0.3	−	−	−	nd

* *The expression of alkB gene was defined in a previous study through the analysis of the activity of the alkB promoter in Rhodococcus sp. strain BCP1 cells transformed with the lacZ gene reporter plasmid, pTP_alkB_LacZ (Cappelletti et al., 2011)*.

# *‘+’, The gene expression is induced and the number of “+” represents the gene expression level; ‘−’, the gene expression is not induced; ‘nd’, not determined*.

Sugars and organic acids in a mixture with *n*-alkanes may result in the inhibition of the *n*-alkanes catabolism by carbon catabolite repression (Rojo, 2009). This is a regulatory mechanism that usually involves regulation at the level of gene expression to prevent transcription of catabolic genes (Rojo, 2009). Here, we demonstrate that the transcription of both *prmA* and *smoA* genes is subject to carbon catabolite repression when alternative substrates are added. In particular, among the tested carbon sources, glucose elicited a complete repression of the transcription of *prmA* and *smoA* (Figure 5). The growth on LB significantly repressed the transcription of *smoA* in the presence of either one of the two inducers (propane or *n*-butane) (Figure 5). The addition of succinate to the medium repressed the transcriptional induction of *prmA* by *n*-butane and partially inhibited the *prmA* and *smoA* transcription induced by propane (by 13− and 8-fold, respectively). These results are taken as evidence for the presence in BCP1 of a catabolite repression mechanism regulating the two *sdimo* genes transcription which depends on the carbon source and type of inducer supplied. On the contrary, the AlkB-like MO coding gene involved in C_6_–C_20_ *n*-alkane oxidation in BCP1 was not subject to catabolite repression by LB, succinate and glucose (Cappelletti et al., 2011). Previous studies on catabolite repression in *Rhodococcus* spp. mainly focused on the metabolism of aromatic compounds (Veselyì et al., 2007; Tomás-Gallardo et al., 2012; Szőköl et al., 2014). In support of the catabolite repression mechanism identified here in BCP1, the analysis of the promoters of the two gene clusters revealed the presence of conserved sequences that share high similarity with the *E. coli* CRP binding site (Figure 7). These conserved sequences were maintained among homologous regions in other Actinobacteria (Figure S5). Additional palindromic sequences were detected upstream of the transcriptional start sites of both the *sdimo* operons (Figures 7A,B). In particular, the palindromic sequence overlapping the −35 region of *prmA* shares similarities with regions described to bind transcriptional regulators belonging to FisR family (Shao et al., 2008). Notably, a member of this family is coded by a gene located immediately upstream of *prm* gene clusters.

Oxygen uptake measurements indicated that the growth of BCP1 on 1-butanol or other metabolism intermediates (butyric aldehyde, butyrate) did not stimulate oxygen consumption after the addition of *n*-butane suggesting their inability to induce the *n*-butane oxidation pathways (Table 2). Conversely, in *Thauera butanovora*, 1-butanol and butyric aldehyde were found to be inducers of *n*-butane oxidation (Sayavedra-Soto et al., 2001, 2005). The induction of O_2_ consumption by BCP1 *n*-hexane-grown cells was observed with the addition of the putative *n*-butane metabolic intermediates (Table 2). This finding suggests that a common enzyme apparatus is involved in the metabolic pathway downstream of the initial oxidation of the two short chain *n*-alkanes (*n*-butane and *n*-hexane). The same indication was provided by the proteomic analysis of the BCP1 soluble fraction. Indeed, the protein patterns induced by growth of BCP1 on *n*-butane and *n*-hexane short-chain *n*-alkanes were similar and distinct from the protein patterns induced by succinate (Figure 8). Among the proteins up-regulated in short-chain *n*-alkanes (SCnA)-grown cells, we identified three alcohol dehydrogenases (DHs) and three aldehyde DHs, which are likely to be involved in the oxidation steps occurring downstream of the monooxygenase hydroxylation (Table S2). Notably, the genes coding for the alcohol DH KDE09894 and for the aldehyde DH KDE09895 are consecutive and associated with *smoABCD* gene cluster in the putative endogenous plasmid pBMC2 (Figure 2). The genes encoding the alcohol DH KDE12275 and the aldehyde DH KDE12276 are also consecutive and in the same genomic region which includes the aldehyde DH KDE12286 and genes for fatty acid metabolism. Two chaperonins both belonging to Cpn60 family chaperone GroEL were also identified in SCnA-grown BCP1 cells (Table S2). In previous studies, Cpn10 chaperonins were shown to be induced in ethene- and ethane-grown NBB4 cells while Cpn 60 chaperon was constitutively expressed (Coleman et al., 2006). The involvement of GroEL chaperone proteins in the bacterial metabolism of small *n*-alkanes has also been reported in strain RHA1 (Sharp et al., 2007) as well as in *Thauera butanovora* and *Methylococcus capsulatus* (Csáki et al., 2003; Kurth et al., 2008).

The isocitrate lyase was strongly expressed in SCnA-grown BCP1 cells (Table S2). This is the key enzyme of glyoxylate bypass that short-circuits the citric acid cycle avoiding the CO_2_-releasing steps and directing acetyl-CoA to conversion to glucose (Cozzone, 1998). The induction of isocitrate lyase in *n*-butane-grow BCP1 cells is in line with the enzymatic activity required to metabolize the intermediates of *n*-butane terminal oxidation pathway, which were revealed by growth and GC assays (Table 1, Figure S2) (Vestal and Perry, 1969). The isocytrate lyase was found to be up-regulated in *n*-hexadecane-grown *A. borkumensis* cells (Sabirova et al., 2006), but not in *M. chubuensis* NBB4 grown on C_2_–C_5_ *n*-alkanes and ethene (Coleman et al., 2011).

Further studies will be aimed at defining regulatory proteins and mechanisms leading to the expression of the different MOs involved in *n*-alkanes oxidation and co-metabolic processes in *Rhodococcus* sp. strain BCP1. Apparently, the study of alkane MOs diversity and regulation is *a priori* necessary for the optimization of microbial transformation of hydrocarbons.

### Conflict of interest statement

The authors declare that the research was conducted in the absence of any commercial or financial relationships that could be construed as a potential conflict of interest.
