# Supplementary material for: Growth of Rhodococcus sp. strain BCP1 on gaseous n-alkanes: new metabolic insights and transcriptional analysis of two soluble di-iron monooxygenase genes
Source: Front Microbiol. 2015 May 12;6:393. doi: 10.3389/fmicb.2015.00393 (PMC4428276; doi:10.3389/fmicb.2015.00393)
Supplement: Supplementary file 1 [file Presentation1.PDF]

Supplementary Material

Growth of *Rhodococcus* sp. strain BCP1 on gaseous *n*-alkanes: new metabolic insights and transcriptional analysis of two soluble di-iron monooxygenase genes

[a] Department of Pharmacy and BioTechnology, University of Bologna, Bologna, Italy

[b] Department of Biological Sciences, University of Calgary, Calgary, Alberta, Canada

[c] Department of Civil, Chemical, Environmental and Materials Engineering, University of Bologna, Bologna, Italy

Correspondence

Dr. Martina Cappelletti
University of Bologna
Department of Pharmacy and BioTechnology
Via Irnerio 42, 40126
Bologna, Italy
e-mail: martina.cappelletti2@unibo.it

Table S1. Primer sets used in this study. The experimental purpose for the use of each primer set is specified.

Target gene	Primer name	Sequence (5' to 3')	Type of analysis
prmA	solmox-For	GTACGGCACCAAGGACCGCC	RT-PCR to verify the *sdimo* clusters co-transcription
	solmox-RT	GAGGGTCTTGCCGTCGTCGC	
prmABCD	iPCR3	TCGACCTCGACCCGAAGAAGAT	
	prmDRev	CTACTGCGCTGTGAGGTCGAA	
smoA	smoAFor	ACTACATCGGTCACAACTCAG	
	smoARev	TCAGATGTCCGTAAACGCGTC	
smoABCD	smoAF1	GAGCGGCACAGCGGTATGAC	
	smoDR1	AGGGCGGCGGTGAGAATGT	
16S rDNA	926F	AAACTYAAAGGAATTGACGG	
	1492R	TACGGYTACCTTGTTACGACTT	
prmA	prmAPE7	CACGACAGCTCGGTGATCTTC	Primer Extension
	prmAPE8	CTTGGTCAGGCTCTGCCTACT	
smoA	prmA2PE1	CTTGGTCGGATACAGTGCGTC	
	prmA2PE2	TGCGTCGCGGTGGAAGTAGCT	
prmA	prmAqPCR-For	CTTGGTCGGATACAGTGCGTC	RT-qPCR
	prmAqPCR-Rev	TACATCGGTCACAACTCAGCA	
smoA	smoAqPCR-For	CTTGGTCGGATACAGTGCGTC	
	smoAqPCR-Rev	TACATCGGTCACAACTCAGCA	
16S rDNA	BCP1-16S-For	ATTAGTGGCGAACGGGTGAG	
	BCP1-16S-Rev	CCCGAGGTCCTATCCGGTAT	

Table S2. Identification of *R.* sp. strain BCP1 proteins induced by the growth on short-chain *n*-alkanes. The peptides univocally identifying one protein are underlined.

Spot ID	Protein name	Genbank Protein ID	M_r	pI	Peptide sequences
**A**	Heat shock protein 60 family chaperone GroEL	KDE12060	56392	4.8	LAGGVAVIK<br>GLNSLADAVK<br>AGAATEVELK<br>NVAAGANPLGLK<br>LLDTAKEVETK<br>AAAPAGDPTGGMGGMDF<br>QIAFNAGLEPGVVAEK<br>KWGAPTITNDGVSIAK<br>EIELEDPYEKIGAELVK<br>TDDVAGDGTTTATVLAQALVR<br>WGAPTITNDGVSIAKEIELEDPYEK<br>IIAFDEEARR<br>AEIEASDSDYDREK<br>VALEAPLKQIAFNAGLEPGVVAEK
	Heat shock protein 60 family chaperone GroEL	KDE12789	55930	4.7	AFGGPTVTNDGVSIAR<br>EIELEDPFENLGAQLVK<br>DAGLDLLGSAR
**B**	Aldehyde dehydrogenase (EC 1.2.1.3)	KDE12286	55109	4.9	ILSYIEIGK<br>IAFTGETTTGR<br>ETLNADIPLAIDHFR<br>LIMQYASQNLIPVTLELGGK<br>VWTNTYHQYPAHAAFGGYK<br>QGDPLDTDTMIGAQASNDQLEK
**C**	ATP synthase beta chain	KDE14110	51981	5.0	FTQAGSEVSTLLGR<br>IGLFGGAGVGK<br>VIDLLTPYVK<br>TVLIQEMITR<br>FTGEPGSVVPLR<br>FLGQNFIVAEK<br>VIGPVVDVEFPR<br>ILEPGIVGAEHFR<br>FTQAGSEVSTLLGR<br>VALSALTMAEYFR<br>MPSAVGYQPTLADEMGELQER
**D**	Aldehyde dehydrogenase (EC 1.2.1.3)	KDE09894	52713	5.2	VGDILSER<br>GQLEALDNGK<br>IAFTGSTEVGK<br>YYAGWATK<br>KVSLELGGK
	Aldehyde dehydrogenase (EC 1.2.1.3)	KDE12276	53166	5.1	GQLEALDNGK<br>IAFTGSTEVGK<br>YYAGWATK<br>KVSLELGGK

**E**	Isocitrate lyase (EC 4.1.3.1)	KDE12029	46785	5.1	TAEGFYGVK<br>LQGTVVEEATLAR<br>EGMTAFVDLQER<br>TAEEIQKDWDTNPR<br>TDAEAATLLTSDVDER<br>AMIAAGVAGSHWEDQLASEK<br>AMIAAGVAGSHWEDQLASEKK<br>AYAPYSDLIWMETGVPDLEVAKK<br>LAADVADVPTVVIAR<br>VLIPTQQHIR<br>AHLDDATIAK<br>QGMTAFVDLQER<br>GSEILWDLVNNEDYINSLGALTGNQAVQQVR<br>EVGAGYFDAIATTVDPNTSTAALK
	Translation elongation factor Tu	KDE12345	43685	5.2	LIQPVAMDEGLR<br>KLLDQGQAGDNVGLLVR<br>TTVTGIEMFRK
**F**	Alcohol dehydrogenase (EC 1.1.1.1)	KDE12333	46477	5.6	ALMGVGAHDIIGVEAK<br>DVGIPDNFGQVR<br>IEYQGVEVVLYDK<br>NMTTVQAADAAVEAAIR<br>DYNVMEAAALYQQEK<br>IEYQGVEVVLYDKVESNPK<br>MAIELNQIWDFPIK
**G**	Alcohol dehydrogenase (EC 1.1.1.1)	KDE12275	38897	5.1	ATISQHSVVK<br>GGTVVITGLANPEK<br>GSLFGSANPQYDIVR<br>YSLEEVNQGYQDLR
	Alcohol dehydrogenase (EC 1.1.1.1)	KDE09895	38535	5.2	ATISQHSVVK<br>GGTVVITGLADPAK<br>AAVLLEPGKPFEIMELDLDGPGVGEVLIK

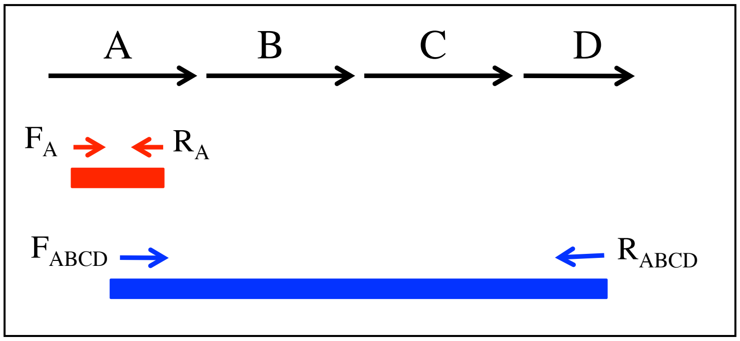

Fig. S1. Schematic representation of the primers used in RT-PCR experiments to analyse the transcriptional organization of each *sdimo* operon (*smoABCD* and *prmABCD*). The primers F_A and R_A were utilized to amplify the hydroxylase subunit gene (gene A) from the total cDNA (solmox-For/solmox-RT for *prmA* and smoAFor/smoARev for *smoA*). The transcription of each gene cluster (A-B-C-D genes) as single polycistronic unit was assessed using the primers F_{ABCD} and R_{ABCD} (iPCR/prmDRev for *prmABCD* and smoAF1/smoDR1 for *smoABCD*). For further details on primer sequences used see Materials and Methods and Table S1.

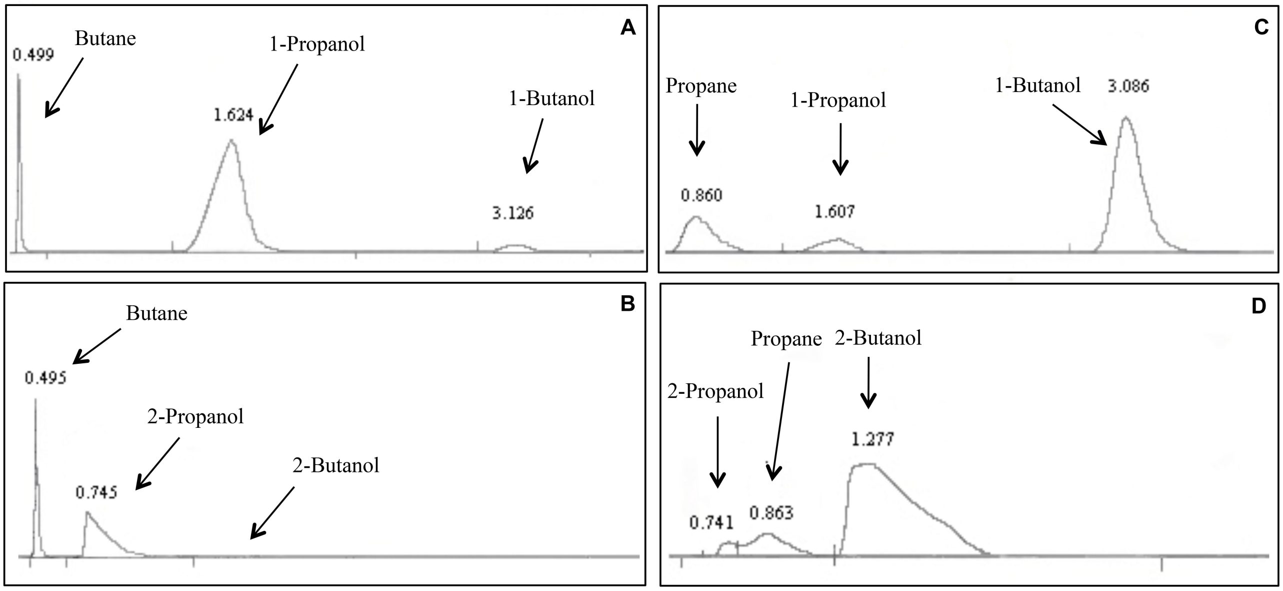

Fig. S2. Representative chromatograms of gas chromatographic (GC) analysis obtained with butane-grown *R.* sp. strain BCP1 cells (A and B) or propane-grown *R.* sp. strain BCP1 cells (C and D) exposed for 15 min to either *n*-butane or propane, respectively, in the presence of 1-propanol (A), 2-propanol (B), 1-butanol (C) or 2-butanol (D). Arrows indicate the oxidation products revealed in each chromatogram. The compounds corresponding to each GC peak are indicated on the basis of pure compounds analysis.

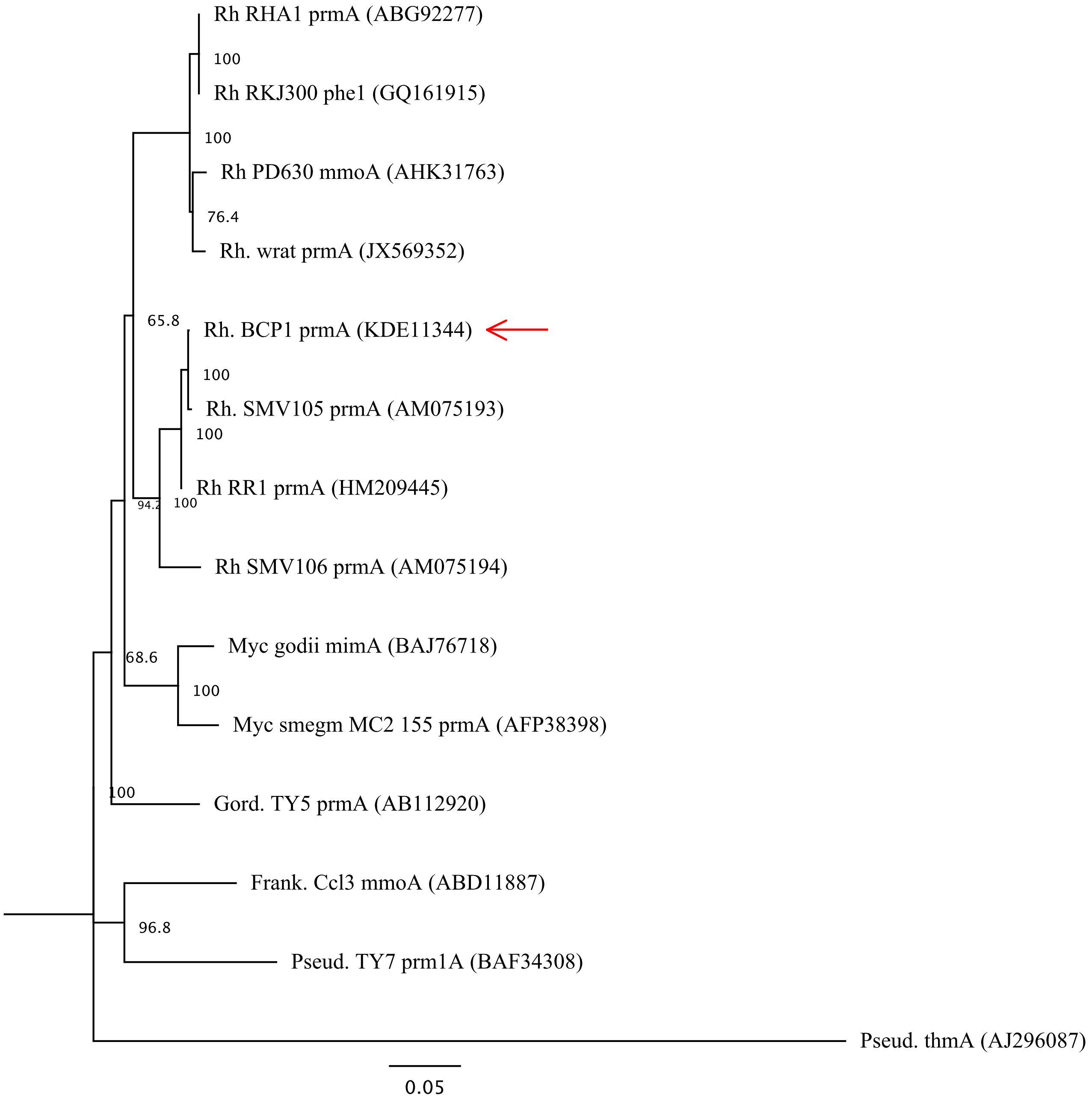

Fig. S3 Phylogenetic tree of *R.* sp. strain BCP1 *prmA* gene. GenBank Accession Numbers of the reference sequences utilized in the analysis are indicated between brackets. *Pseudonocardia tetrahydrofuranoxydans thmA* gene, coding for tetrahydrofuran monooxygenase, was used as outgroup. Bootstrap values based on 1000 replications are listed at the branch nodes. Abbreviations: Rh., *Rhodococcus*, Myc., *Mycobacterium*, Frank., *Frankia*, Pseud., *Pseudonocardia*, Gord., *Gordonia*; wrat., *wratislaviensis*; smegm., *smegmatis*.

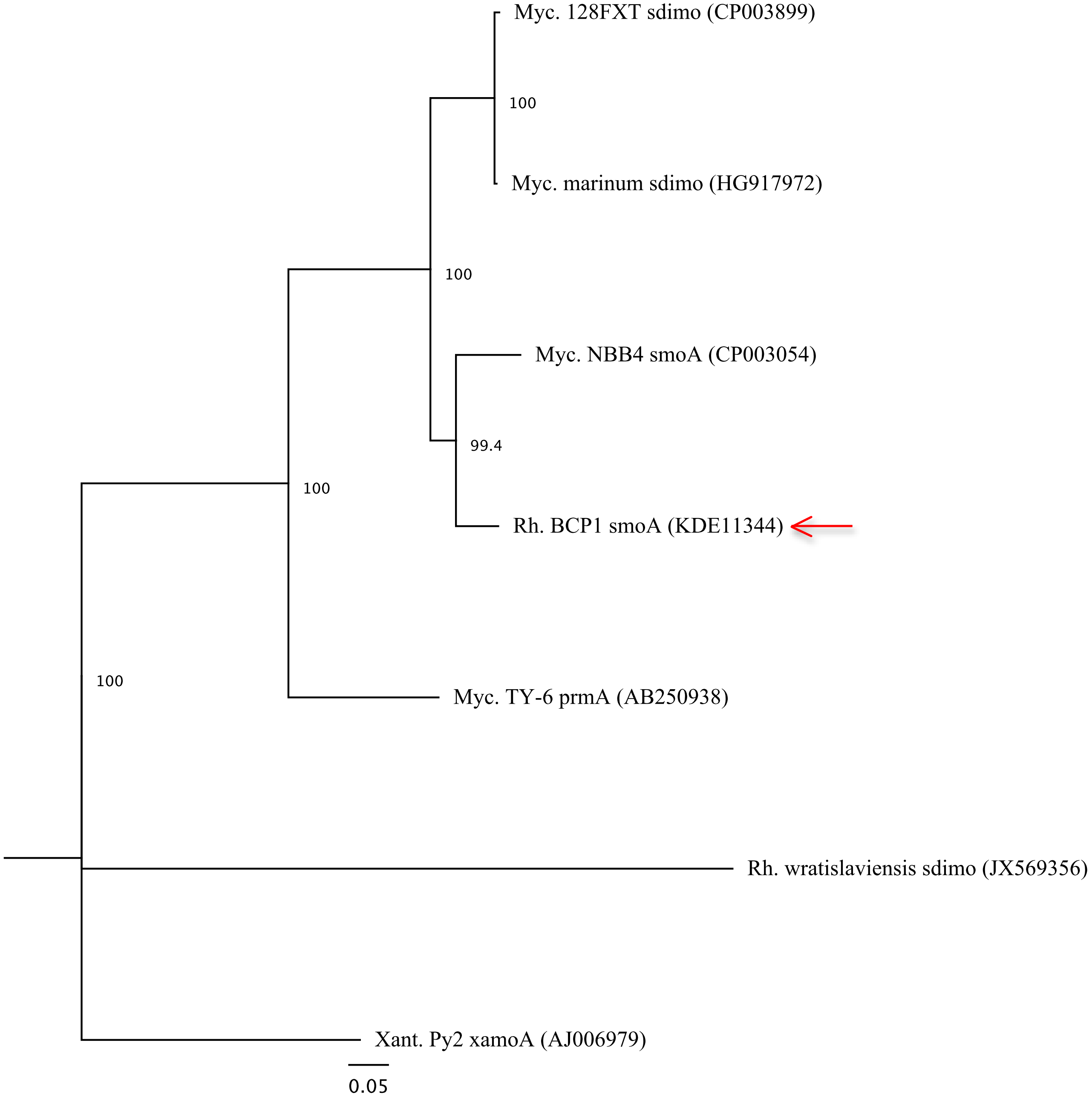

Fig. S4 Phylogenetic tree of *R.* sp. strain BCP1 *smoA* gene. Phylogenetic tree of *R.* sp. strain BCP1 *smoA* gene. GenBank Accession Numbers of the reference sequences utilized in the analysis are indicated between brackets. *Xanthobacter* Py2 *xamoA* gene, coding for an alkene monooxygenase, was used as outgroup. Bootstrap values based on 1000 replications are listed at the branch nodes. Abbreviations: Rh., *Rhodococcus*, Myc., *Mycobacterium*, Xant., *Xanthobacter*.

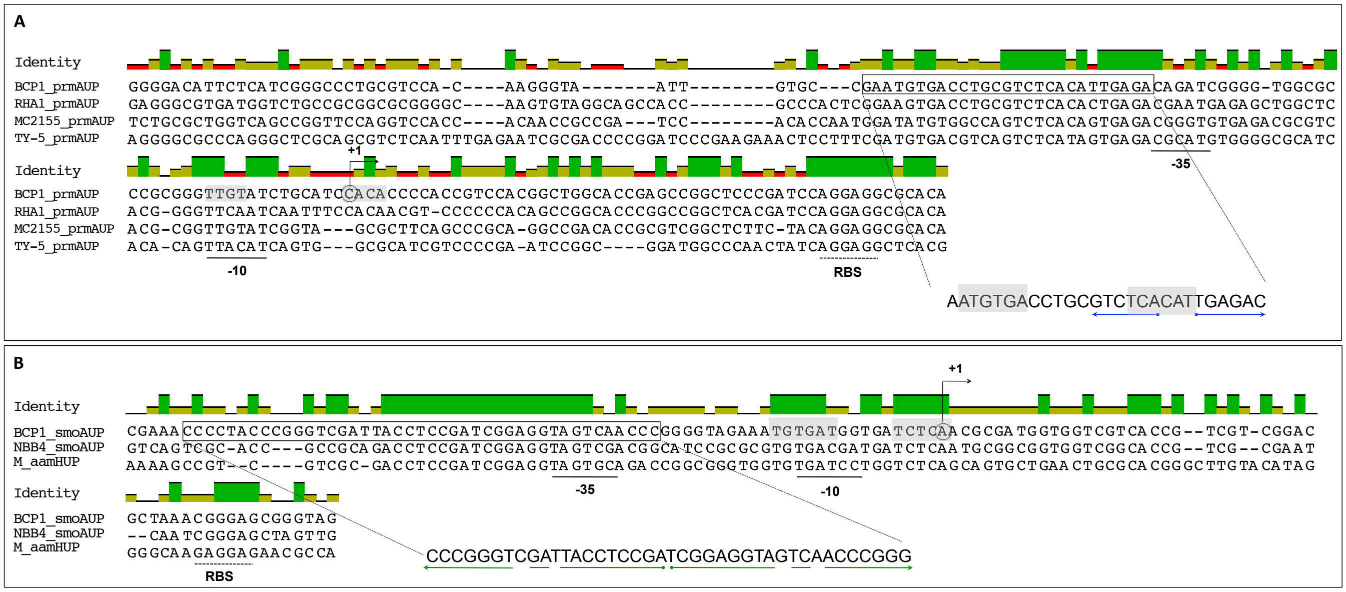

Fig. S5 Alignment of *R.* sp. strain BCP1 *prmA* (Panel A) and *smoA* (Panel B) promoters with the promoter regions of homologous genes (those presenting the entire nucleotide sequence in database). The locus tags of the genes downstream of the promoters taken into consideration are as follows: RHA1_ro00441, *R. jostii* RHA1; MSMEI_1927, *M. smegmatis str. MC2 155*; AB112920, *G.* strain sp. TY5; Mycch_5395, *M. chubuensis* NB400; MMAR_0131, *M. marinum* M. The transcriptional start sites detected by primer extension experiments for each *sdimo* are circled. The deduced putative -10 and -35 promoter regions as well as the putative ribosome-binding sites (RBS) are indicated below the alignments. The partially conserved regions, including inverted repeat sequences described in the text are shown inside the black boxes (and in the enlarged section). The nucleotides forming the inverted repeats are indicated with divergently oriented arrows [each inverted repeats are indicated with arrows with different colours (blue or green)]. Putative CRP (Catabolite Repression Protein) binding sites present in BCP1 sequences are shaded in grey.
